# Dry Cupping

**Published:** 1852-07

**Authors:** 


*Dry Cupping.* By Dr. B. H. Washington, of Woodburn, Ky.

Dr. Bowling: Please to allow me, through your columns, respectfully to invite the attention of the profession to a few items I have had the good fortune to stumble upon in my practice.

Having heard dry-cupping on the spine very highly recommended for its anodyne, alterative, and emmenagogue effects, by the late Dr. Prather, of St. Louis, and having seen its remarkable effects in his hands, I have used it freely and extended its use, and have never been able to hear or read of a superior remedy, though the last ten years have not been passed in idleness.

As an anodyne, it relieves pain without checking any of the secretions; but on the contrary, it regulates the whole system, and brings every organ to the normal standard.

About five years since, a negro man cut his leg severely with a broad-axe, midway the tibia. The wound was dressed in the usual manner; but the next morning his wife informed me he had not slept a wink the whole night. There being some eight or ten children in the cabin, I thought it best not to dry-cup him until night. In half an hour after he was dry-cupped, he fell asleep, never moved the whole night, and awoke next morning free from pain. The cupping was repeated every alternate night; freedom from pain was the result, and the leg soon healed. In almost every case of bruise or wound, I have used it with signal benefit; and, from my experience, have not the slightest doubt that, if it was added to the usual water-dressing, at least eight-tenths of the cases where mortification now occurs, could be healed without any such result. Many cases of its anodyne effects could be detailed, but one more will suffice. When called to see a negro woman, found her sitting up in bed; great difficulty in breathing; violent pain in left side, greatly increased on drawing a full breath, or coughing; tongue dry; pulse 125, hard and full; was informed she had had an attack of pleurisy about a year previous, and that the pain was in precisely the same spot. Independent of an auscultation, there was something in her looks and actions that convinced me it was not an inflammatory case. I took from a table a large tumbler, and applied successively nearly the whole length of the spine. When I was done, she said she was nearly well. To prevent the pain returning, and to assist in arousing the skin, two grains quinine and two of cayenne pepper were administered, and she was directed to have herself sponged with warm water from head to foot. Next day she was able to walk about well.

Having been dry-cupped a few days after a dislocated shoulder was reduced, I attempted to raise my arm to my head, while the cup was over the origin of the brachial nerves, and found my arms partially paralyzed. It immediately occurred to me that it would be a most excellent remedy for counteracting the resistance of the muscles in cases of dislocation. About six months afterwards, a boy aged seven years was thrown from a horse, and having thrown out his arm to save himself, dislocated the elbow joint. The dry-cup was applied over the origin of the brachial nerves, and the arm was easily reduced, apparently without much suffering; the water-dressing was applied, and the patient soon recovered. In my opinion, if the dry-cup was applied over the origin of the nerve distributed to the dislocated part, instead of the patient being nearly pulled to pieces with ropes and pulleys, as is sometimes the case, the joint could be reduced with far less suffering, and much easier.

Of the alterative effects of dry-cupping, I scarcely know where to begin detailing cases; it is incomparably superior to blue pill or any form of mercury. In January, 1848, I took charge of the case of a woman with chills, who had been in the hands of a distinguished physician for about six months. For the purpose of invigorating her health, so that the chills would *stay stopped*, dry cupping, with frequent sponging of the whole body with tepid water, was recommended. As a matter of course, the chills were cured, and, moreover, have never returned since. This case is mentioned, not because of the thorough cure of the chills, for in that there was nothing uncommon, but for another consideration. When put in charge of the case, I was informed she had had a tetter on her leg for thirty years, and a great number of physicians had prescribed for her without success. I concluded not to do any thing for the tetter until she was cured of the chills; but to my great surprise the dry-cupping had cured that also, and so thoroughly that it has never returned. The cupping was continued every alternate night for about four months. I took the hint thus accidentally given, and have since cured a case of tetter of twenty years' duration, and it continues well; it is now about three years since the cure. Of course, I now recommend dry-cupping in preference to all other remedies, for tetter. If you wish to see the alterative effects of dry-cupping, the first time you ride out to Mill Creek, near your city, call on Mr. Edward H***. In '47 he was not able to walk across the room without a crutch, from rheumatism and an injury in the groin. For about four years he has been walking without his crutch, briskly, too, and can ride on horseback anywhere, while in '47 he could not ride on horseback at all; and a few weeks since he told me that he was in better health than he had been for twenty years. The course of treatment recommended was dry-cupping the whole length of the spine, with frequent sponging of the whole body with warm water. For ulcers it is superior to all salves and ointments, and even superior to the water-dressing.

For its emmenagogue effects, dry-cupping can be recommended with equal confidence. I do not believe any case of amenorrhœa or dysmenorrhœa would resist its steady application, accompanied with frequent bathing. Some years since, I had a severe case of dysmenorrhœa, of only five months' duration, however. Feeling anxious to afford prompt relief, a celebrated physician was consulted, but not taking his plan, concluded to try dry-cupping, as I had three weeks to go upon. The cups were applied every alternate night the whole length of the spine, more strongly over the origin of the nerves distributed to the uterus; and the result was, at the next period, only a slight headache for a few hours was felt, and the second period no inconvenience whatever.

The glasses commonly used for cupping are too small—tumblers with a thick rim answer much better. Each one should stay on about five or ten minutes, and when it is desirable to produce an impression on a given part, the cup should be more strongly applied over the origin of the nerves distributed to that part. With nervous patients only one or two cups should be applied at first, afterward gradually increasing the number. If they are applied the whole length of the spine at first, the next day perhaps the patient will not be able to hold his hands still. Like all other remedies, it requires judicious use.

I hope the reader will not consider dry-cupping my favorite hobby; I have one I ride in preference. To any one disposed to verify the above statements, names, dates, and residence will be given on application to me. *Nashville Journal of Medicine.*

